# When approximate number acuity predicts math performance: The moderating role of math anxiety

**DOI:** 10.1371/journal.pone.0195696

**Published:** 2018-05-02

**Authors:** Emily J. Braham, Melissa E. Libertus

**Affiliations:** Department of Psychology and Learning Research and Development Center, University of Pittsburgh, Pittsburgh, Pennsylvania, United States of America; Mälardalen University, SWEDEN

## Abstract

Separate lines of research suggest that people who are better at estimating numerical quantities using the approximate number system (ANS) have better math performance, and that people with high levels of math anxiety have worse math performance. Only a handful of studies have examined both ANS acuity and math anxiety in the same participants and those studies report contradictory results. To address these inconsistencies, in the current study 87 undergraduate students completed assessments of ANS acuity, math anxiety, and three different measures of math. We considered moderation models to examine the interplay of ANS acuity and math anxiety on different aspects of math performance. Math anxiety and ANS acuity were both unique significant predictors of the ability to automatically recall basic number facts. ANS acuity was also a unique significant predictor of the ability to solve applied math problems, and this relation was further qualified by a significant interaction with math anxiety: the positive association between ANS acuity and applied problem solving was only present in students with high math anxiety. Our findings suggest that ANS acuity and math anxiety are differentially related to various aspects of math and should be considered together when examining their respective influences on math ability. Our findings also raise the possibility that good ANS acuity serves as a protective factor for highly math-anxious students on certain types of math assessments.

## Introduction

In today’s society, it is becoming increasingly important for people to be able to make sense of mathematical information. Yet, many adults struggle when using math in everyday situations, such as choosing the best deal on a cell phone contract or interpreting statistics to make informed decisions about their own health. What factors contribute to difficulties with mathematics? Do these factors interact to influence math performance and if so, how? The present paper will explore two specific factors: math anxiety and the foundational understanding of quantities that is at the core of the approximate number system.

A substantial body of research attributes low math performance to math anxiety—a negative emotional reaction to situations involving numbers or math [1–3]. Math anxiety can be defined as ‘a feeling of tension, apprehension, or fear that interferes with math performance’ [4]. Importantly, math anxiety is a distinct construct from generalized anxiety and it explains variance in math performance over and above general test anxiety [2]. A negative correlation between math anxiety and math performance emerges for children during the elementary or middle school years [5–7], and as children develop, their math anxiety becomes more specific and distinct from generalized anxiety [8]. In adults, math anxiety is associated with decreased performance on math assessments that vary in complexity; adults with high levels of math anxiety perform worse not only on math assessments that involve complex math equations, but also on simple tasks that involve reading math problems aloud and deciding which of two symbolic numbers is larger [4,9–11]. Adults with high levels of math anxiety also tend to avoid math courses and endorse negative beliefs about their own math abilities [2], in turn strengthening the association between math anxiety and math performance.

The exact mechanisms by which math anxiety affects math performance remain unclear. On the one hand, some researchers suggest that math anxiety negatively affects math performance by consuming cognitive resources, such as attention and working memory [12]. When highly math anxious people focus their attention towards the worrisome thoughts, it may be more difficult for them to maintain focus on the math assessment and their performance may suffer due to limited working memory resources. On the other hand, other researchers propose that math anxiety may be due to basic number processing deficits [3]. Compared to their peers with low math anxiety, adults with high math anxiety even perform worse on basic number comparison tasks in which they have to identify the larger of two Arabic numerals [10]. In addition, adults with high math anxiety also perform worse on simple counting tasks. This difference in counting performance is thought to result from differences in working memory suggesting that the two accounts may not be entirely independent [9].

In the cognitive literature, recent research focuses on linking people’s math difficulties to how they process numerical quantities (for reviews, see [13,14]). One system for processing numerical quantities, the approximate number system (ANS), can be used to make intuitive estimates and comparisons about the number of items in collections without counting. Mental representations of quantities in the ANS are independent of language or an understanding of symbols: they are present in non-verbal infants and in non-human animals [15–18]. These representations are imprecise and have substantial overlap with their neighboring quantity representations. The degree of overlap increases with larger quantities, i.e., smaller quantities are represented more precisely than larger quantities, and discriminability of any two ANS representations is a function of the ratio between them [19].

Importantly, ANS precision varies across people. For example, adults with more precise approximate quantity representations may easily detect differences between quantities in a 1.11 ratio (e.g., comparing 20 and 18 objects), while others may struggle to discriminate quantities in a 1.33 ratio (e.g., comparing 20 and 15 objects). While some studies find individual differences in ANS acuity to relate to math performance (e.g., [20–25]), others report null or mixed results (e.g., [26–28]).

With the exception of a handful of recent studies, research linking ANS acuity to math performance has largely ignored the emotional components associated with performing math. How might ANS acuity and math anxiety relate? In a sample of undergraduate students, Lindskog and colleagues [29] found that students with higher math anxiety had lower ANS acuity. Importantly, math anxiety fully mediated the association between ANS acuity and math performance. In their discussion of the results, Lindskog and colleagues [29] raise the possibility that math anxiety has deep roots in the earliest system for processing numerical information: the ANS. As children learn to represent numbers symbolically through counting and Arabic numerals, they draw on their ANS representations [30]. According to the proposed theory, a core deficit in ANS acuity could put children at risk for difficulty when they first learn symbolic numbers, which could create negative feelings about mathematics and lead to math avoidance and diminished performance [29].

However, counter to Lindskog and colleagues’ proposed theory, three recent studies did not find evidence of an association between ANS acuity and math anxiety in samples of adults [31] or children [32,33]. Based on the mixed results in the literature [29,31–33] there is clearly a need to further investigate the possible accounts of the relation between ANS acuity, math anxiety, and math performance. It is possible that math anxiety and ANS acuity develop along separate trajectories, and potentially interact to influence math performance. For example, the association between ANS acuity and math performance could change with varying levels of math anxiety. Yet, this alternative theory has not been fully tested: in two of the previous studies the relation between ANS acuity and math anxiety was not the primary focus [32,33] and the third lacked a measure of math performance [31].

In order to better understand the complex integration of factors driving differences in people’s mathematical abilities, we examined the relation between ANS acuity and math anxiety in a sample of undergraduate students and tested both mediation and moderation models to examine the interplay of ANS acuity and math anxiety on three different outcome measures of math performance (written calculation, math fluency, and quantitative reasoning). We had no specific hypotheses regarding the relation of ANS acuity and math anxiety for each of the three aspects of math. However, we decided to assess all three to explore possible differences in their respective relations with ANS acuity and math anxiety.

## Method

### Participants

Eighty-seven undergraduate students (41 males) participated in a 1-hour laboratory study in exchange for course credit. The average age of the students was 20.33 years (*SD* = 5.12). This study was approved by the University of Pittsburgh Institutional Review Board and all students provided written, informed consent prior to participation. Data from one student were excluded from all analyses due to experimenter error, resulting in a final sample of 86 students.

### Measures

#### ANS acuity

To measure students’ ANS acuity, we presented them with briefly flashing arrays of blue and yellow dots on a computer screen and asked them to select the more numerous array as quickly and as accurately as possible. Students responded by pressing a corresponding color-coded key. The color of the correct response was counterbalanced across trials. The images, extracted from the freely available Psychological Assessment of Numerical Ability (Panamath; www.panamath.org), were displayed using a custom-made MATLAB script. Each colored array of dots appeared on a gray background and contained between 12 and 36 dots of various sizes (average dot diameter = 36 pixels; allowed variation = 20%).

A total of 360 trials were equally distributed across four blocks, varying in presentation format: (Block 1) arrays presented simultaneously with spatial separation (i.e., yellow dots on the left and blue dots on the right half of the screen appeared at the same time), (Block 2) arrays presented simultaneously with spatial overlap (i.e., yellow and blue dots both clustered in the middle of the screen appeared at the same time), (Block 3) arrays presented sequentially with spatial separation (i.e., yellow dots on the left and blue dots on the right half of the screen appeared one followed by the other), and (Block 4) arrays presented sequentially with spatial overlap (i.e., yellow and blue dots clustered in the middle of the screen appeared one followed by the other). Block order was counterbalanced across students.

Each trial started with a fixation cross (500 ms), followed by the arrays of blue and yellow dots (in simultaneous blocks, both arrays appeared for 1500 ms; in sequential blocks, one array appeared for 750 ms followed by the other for 750 ms), followed by a blank screen. Students were able to respond either during the presentation of the dot arrays or during the blank screen. The difficulty of the trial depended on the ratio between the numbers of dots, i.e., the larger number of dots divided by the smaller. Within each block, there were 18 trials for each of five ratio categories: 1.11 (e.g., 10 blue dots: 9 yellow dots), 1.14, 1.2, 1.25, and 1.33. To ensure that students relied on numerical information rather than other perceptual cues, we varied the total surface area of each array across trials. Within each block, there were 30 trials for each of three cumulative area types: Congruent (i.e., the array with the larger number had the larger cumulative area), Incongruent (i.e., the array with the smaller number had the larger cumulative area but both arrays had equal cumulative perimeter), and Neutral (i.e., the arrays had equal cumulative area).

For each block, ANS acuity was measured as the proportion of correct responses (i.e., accuracy). Accuracy has been shown to be a more reliable measure of ANS acuity compared to Weber fractions (i.e., an index of the imprecision of participants’ ANS representations) or ratio effects in accuracy or response time [34]. Following the Jolicoeur method [35], trials in which a student’s response times were above or below 2.5 standard deviations from their average response time were discarded as outliers (on average, 7.5 out of 360 trials). Due to computer errors, two students did not complete Block 3 and one student did not complete Block 4. Missing data were not interpolated.

#### Math abilities

Students’ math abilities were assessed using three subtests from the nationally normed Woodcock Johnson III Tests of Achievement [36]: Calculation, Math Fluency, and Applied Problems. The *Calculation* subtest was used to measure students’ ability to perform mathematical computations in traditional written format. The problems included arithmetic, as well as geometric, trigonometric, logarithmic, and calculus operations. The *Math Fluency* subtest measured speeded mental arithmetic and required students to solve simple addition, subtraction, and multiplication problems within a 3-minute time limit. Students were told to start with the first item and work as quickly as possible without making mistakes. The *Applied Problems* subtest measured the ability to listen to an orally presented word problem, select the relevant information, recognize the procedure, and perform the appropriate calculations. The experimenter read the word problems aloud to the student, but the student also had access to at least some of the information in the form of a written word problem, a labeled figure, or both. Students gave verbal responses, but were allowed to use scratch paper if needed. One Applied Problems score could not be calculated due to time constraints.

#### Math anxiety

Math anxiety was measured using a 30-item version of the Mathematics Anxiety Rating Scale [37]. Each item on the questionnaire described a situation involving mathematics (e.g., “Figuring the sales tax on a purchase that costs more than $1.00”; “Taking the mathematics section of a college entrance exam”). Students reported their level of anxiety in each situation on a 5-point scale (1 = *not at all anxious*; 5 = *very anxious*). Each student’s math anxiety score was calculated as the average of all 30 items. Three participants did not complete the math anxiety questionnaire due to time constraints.

#### Working memory (WM) span

As a measure of students’ verbal working memory, we administered a widely used backward digit span task. In this task, the students listened to pre-recorded digit sequences (e.g., “3, 9, 7”) at a rate of one item per second. After each sequence, they had to recall the sequence in reverse order. The possible sequence lengths ranged from three to twelve items and for each sequence length there were two trials. Administration continued until the student failed on both trials at a particular sequence length. The span score was marked as the length of the longest sequence in which the student recalled at least one of the trials correctly. Span scores for two participants could not be calculated due to experimenter error.

#### Math courses

On a demographics questionnaire, students were asked to self-report the number of college-level math courses they had taken as a proxy for math content knowledge. No participants reported repeating the same course. Twenty students took no math courses, 40 students took one course, 18 students took two courses, and 8 students took three or more courses. One student chose not to report the number of math courses taken.

### Procedure

All participants completed the measures in the following order: 1) ANS acuity task, 2) working memory test, 3) math ability assessments, 4) math anxiety questionnaire, 5) demographics questionnaire. We administered the math anxiety questionnaire after the math assessments in order to minimize the influence of the questionnaire on the students’ math performance and to avoid creating a stereotype threat experience that could inflate students’ situational levels of anxiety, stress, or arousal during the math assessment [38–40].

## Results

### Preliminary analyses

An initial examination of the ANS task revealed similar accuracy scores across the four blocks (Block 1: *M* = .77, *SD* = .07; Block 2: *M* = .69, *SD* = .07; Block 3: *M* = .77, *SD* = .06; Block 4: *M* = .73, *SD* = .08) and highly significant intercorrelations (Block 1 and Block 2: *r* = .39, *p* < .001; Block 1 and Block 3: *r* = .42, *p* < .001; Block 1 and Block 4: *r* = .38, *p* < .001; Block 2 and Block 3: *r* = .36, *p* = .001; Block 2 and Block 4: *r* = .49, *p* < .001; Block 3 and Block 4: *r* = .55, *p* < .001). Both the Kaiser-Meyer-Olkin measure of sampling adequacy (.73) and Bartlett’s test of sphericity (*χ*^*2*^(6) = 78, *p* < .001) suggested the data were suitable for a principle components analysis. The scree plot and eigenvalues indicated that the data could best be described by one component which explained 58% of the variance. This analysis suggested that the different blocks of trials were measuring the same underlying construct, and thus we used the factor score in all further analyses to obtain a reliable estimate of ANS acuity irrespective of presentation format. Further, combining the four ANS task blocks resulted in a total of 360 trials, which is around the number of trials necessary to obtain acceptable levels of reliability on an ANS task [41]. Note that the pattern of results for all analyses is the same if average accuracy scores are used instead of the factor score.

### Correlations

First, we examined correlations between the ANS acuity factor score and math anxiety with each of the three math assessments (Calculation, Math Fluency and Applied Problems). Descriptive statistics and bivariate correlations among all variables are displayed in Table 1. Students’ ANS acuity correlated positively with Math Fluency and Applied Problems scores, while math anxiety correlated negatively with only Math Fluency. Correlational results between performance on each of the blocks in the ANS acuity task, math anxiety, and math performance can be found in the Supplemental Information.

**Table 1:** Descriptive statistics and pearson correlations between all measured variables.

			Correlations					
	*M*	*SD*	1	2	3	4	5	6
1. Math Courses	1.28	1.25						
2. WM Span	4.94	1.68	-.27*					
3. ANS Factor Score	0	1	-.14	.20^†^				
4. Math Anxiety	2.48	.60	-.09	-.13	-.04			
5. Calculation	34.3	5.38	.26*	.14	.01	-.19^†^		
6. Math Fluency	127.91	21.79	-.06	.22*	.27*	-.27*	.26*	
7. Applied Problems	53.79	4.49	.06	.21^†^	.27*	-.22^†^	.48**	.40**

Note.

^†^*p* < .10.

*p < .05.

**p < .001.

The average accuracy score across the four ANS blocks was .74 (*SD* = .05). ANS acuity is presented in the table as a factor score which has a mean of 0 and standard deviation of 1 by construction.

### Main-effects models

Second, we used linear regression to examine the unique contributions of ANS acuity and math anxiety for predicting each of the subtests of math ability (Table 2, Model 1). Given evidence highlighting the role of working memory in the association between math anxiety and math ability [12], we included working memory capacity as a covariate. As a proxy for students’ experience with formal mathematics, we also included students’ self-reported number of college math courses as a covariate. College math courses, working memory span, and math anxiety were centered around the mean in order to reduce multicollinearity and aid interpretation [42].

**Table 2:** Main effects and interaction models predicting Woodcock Johnson math scores (Calculation, Math Fluency, and Applied Problems), controlling for number of college math courses and working memory span.

	Model 1 *B(SE B)*, *p*	Model 2 *B(SE B)*, *p*
**Predicting Calculation Scores**		
College Math Courses	**1.33(.49), .008** **	1.34(.49), .008**
Working Memory Span	.65(.37), .082^†^	.59(.37), .114
ANS Acuity	.04(.60), .945	-.05(.60), .934
Math Anxiety	-1.24(.98), .21	-1.28(.98), .192
ANS Acuity x Math Anxiety		1.46(1.08), .175
**Predicting Math Fluency Scores**		
College Math Courses	-.13(1.96), .949	-.14(1.97), .944
Working Memory Span	1.77(1.47), .235	1.86(1.49), .216
ANS Acuity	5.10(2.38), .036*	5.24(2.41), .033*
Math Anxiety	-8.85(3.92), .027*	-8.77(3.94), .029*
ANS Acuity x Math Anxiety		-2.37(4.35), .587
**Predicting Applied Problems Scores**		
College Math Courses	.47(.41), .246	.49(.40), .224
Working Memory Span	.47(.31), .128	.39(.30), .195
ANS Acuity	1.12(.49), .026*	1(.49), .043*
Math Anxiety	-1.28(.81), .118	-1.34(.79), .094^†^
ANS Acuity x Math Anxiety		1.91(.88), .032*

Note.

^†^*p* < .10.

*p < .05.

**p < .001.

#### Calculation

Model 1 explained 14% of the variance in Calculation scores, *F*(4,74) = 2.92, *p* = .027. Number of college math courses was significantly associated with Calculation scores over and above ANS acuity, math anxiety, and working memory.

#### Math Fluency

Model 1 explained 16% of the variance in Math Fluency scores, *F*(4,74) = 3.5, *p* = .011. ANS acuity and math anxiety were both significantly associated with Math Fluency scores over and above the remaining variables.

#### Applied Problems

Model 1 explained 15% of the variance in Applied Problems scores, *F*(4,74) = 3.26, *p* = .016. ANS acuity uniquely predicted Applied Problems scores over and above the other variables.

### Mediation models

Third, we planned to run mediation analyses, paralleling those of Lindskog and colleagues [29]; however, we did not find a significant correlation between ANS acuity and math anxiety (Table 1), precluding us from running any mediation analyses.

### Moderation models

Finally, in order to test an alternative model and examine if the relation between ANS acuity and math ability varies as a function of math anxiety level we added an interaction term between ANS acuity and math anxiety to the linear regression models (Table 2, Model 2).

#### Calculation

Model 2 explained a significant amount of the variance in Calculation scores (16%), *F*(5,73) = 2.74, *p* = .025. Number of college math courses remained a significant predictor of Calculation scores after additionally controlling for the interaction term. Neither ANS acuity nor math anxiety was uniquely related to Calculation scores, and the interaction between ANS acuity and math anxiety did not reach statistical significance either.

#### Math Fluency

Model 2 explained a significant amount of the variance in Math Fluency scores (16%), *F*(5,73) = 2.84, *p* = .02. ANS acuity and math anxiety both remained unique significant predictors, but the interaction between ANS acuity and math anxiety did not reach statistical significance.

#### Applied Problems

Model 2 explained a significant amount of the variance in Applied Problems scores (20%), *F*(5,73) = 3.70, *p* = .005. The addition of the interaction term in Model 2 over Model 1 accounted for an additional 5% of the variance, *R*^*2*^ change = .05, *F*(1,73) = 4.77, *p* = .032, and the interaction term between ANS acuity and math anxiety was indeed significant.

**Fig 1:**
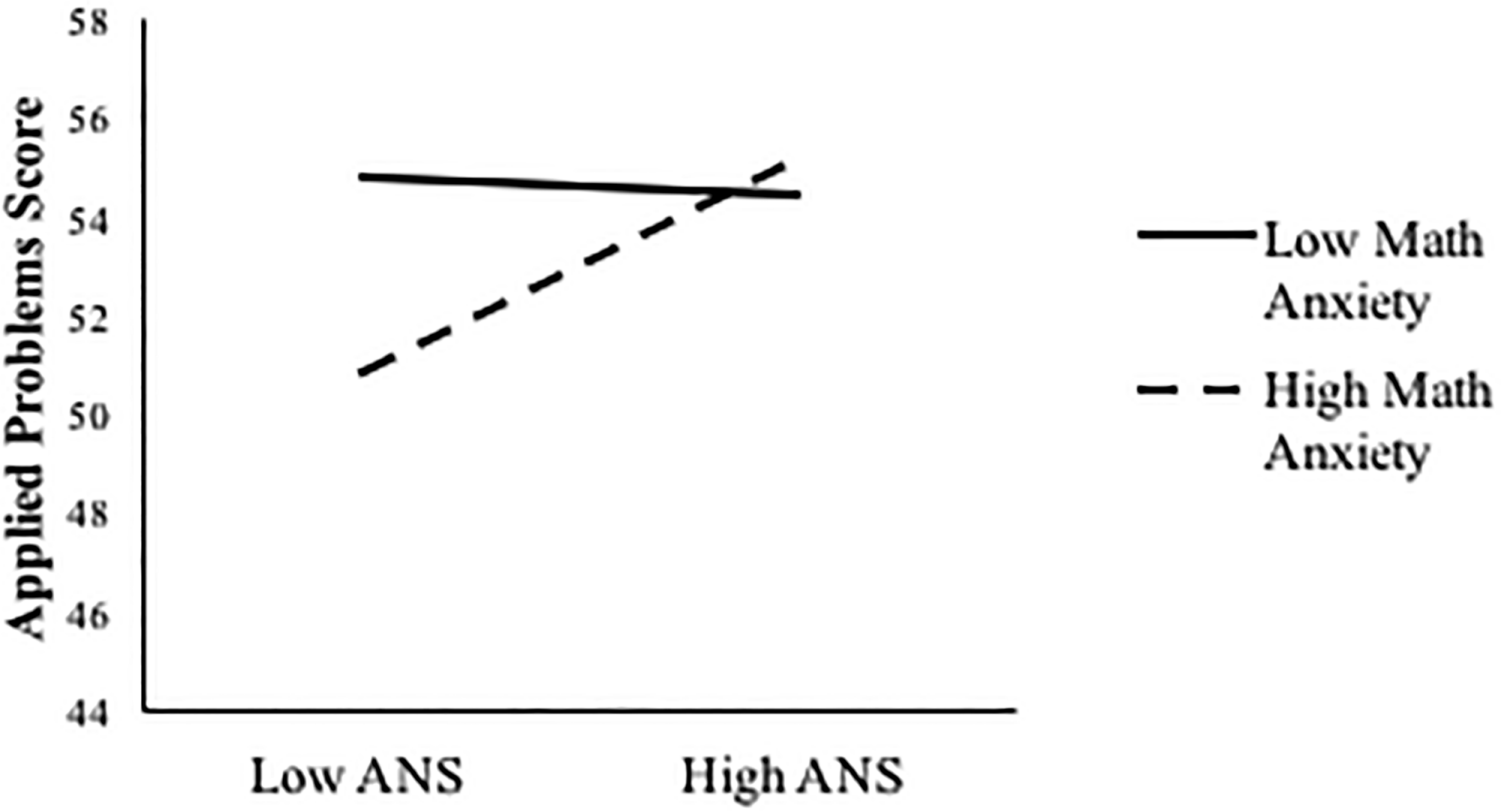
Applied Problems scores plotted as a function of math anxiety and ANS acuity. Students with relatively higher math anxiety showed a pronounced positive relation between ANS acuity and Applied Problems scores. Note that ANS acuity and math anxiety are treated as continuous variables, but are plotted at 1 SD above the mean and 1 SD below the mean for visual purposes.

A visual representation of the interactive effects of ANS acuity and math anxiety on Applied Problems scores, controlling for number of college math courses and working memory span, is presented in Fig 1. Simple slopes of ANS acuity on Applied Problems are plotted at two different levels of math anxiety (High: 1 SD above the mean, Low: 1 SD below the mean). Simple slopes post-hoc analyses were used to examine if the slopes at each level of math anxiety were significantly different than 0. At high levels of math anxiety, the simple slope of ANS acuity on Applied Problems scores was in a positive direction and significantly different than zero, *t*(85) = 3.18, *p* = .002. However, at low levels of math anxiety the simple slope of ANS acuity on Applied Problems was not significantly different than zero. This suggests that the positive association between ANS acuity and Applied Problems scores is only present in individuals with high math anxiety. Thus, it appears that the relation between ANS acuity and Applied Problems scores changes with varying levels of math anxiety.

## Discussion

Most research on the role of ANS acuity and math anxiety for math ability has examined these factors in isolation. In the few exceptions, mixed results are reported about the relation between ANS acuity and math anxiety, and their influence on math performance [29,31–33]. The present study yielded four main findings, which we will discuss in more detail below. First, we did not find a significant correlation between ANS acuity and math anxiety. Second, math anxiety was a unique significant predictor of only one math assessment: Math Fluency. Third, ANS acuity was a unique significant predictor of both Math Fluency and Applied Problems. Finally, there was a significant interaction between math anxiety and ANS acuity on Applied Problems.

### ANS acuity and math anxiety

In the current study, we did not find a significant correlation between ANS acuity and math anxiety. These findings are in line with three other studies with children and adults [31–33]. As suggested by Maloney and colleagues [9,10], math anxiety could be the result of poor basic number processing skills such as deficits in enumeration and symbolic number comparison. However, in the present study we do not find any evidence for a link between math anxiety and non-symbolic number comparison (see Table 1). Thus, while math anxiety may be linked to some basic number processing skills, it may affect those requiring an understanding of number symbols more so than non-symbolic number processing skills. Since we did not assess any basic symbolic number skills, it is impossible for us to test this hypothesis.

Our results also contradict those by Lindskog et al. [29], who found that math anxiety and ANS acuity are correlated in adulthood and suggested that math anxiety may be due to poor ANS acuity. Lindskog et al. used an ANS task in which dots were presented simultaneously in an intermixed format. In our study, even if we examine the correlation between math anxiety and only the ANS block that is most similar in format (Block 2; see Supplemental Information for correlations) we do not find a significant association. It is possible that the differences between the studies are due to the variability of participants’ responses on the math anxiety questionnaire. Lindskog et al. report a mean math anxiety score of 17.4 with a standard deviation of 11.5 (66% of the mean), while our standard deviation is only 24% of the mean. Future research is needed to replicate our findings in participants with a wider range of math anxiety levels.

Our findings support the theory that math anxiety and ANS acuity may develop along separate trajectories and that math anxiety may be more likely the result of environmental influences (for review, see [43]). For example, children’s math anxiety can develop as a product of the home or classroom environment through social interactions with math-anxious parents [44] or math-anxious elementary school teachers [45]. Children’s math anxiety can also be reduced through high-quality parent support at home [46]. Thus, it seems likely that students with poor ANS acuity may have high or low math anxiety, and students with good ANS acuity may have high or low math anxiety. The interesting question then becomes, what are the unique influences of each factor and how do they interact?

### Math anxiety and math performance

We found that math anxiety was a unique significant predictor of students’ ability to perform speeded mental arithmetic (Math Fluency), but not mathematical computations in traditional written format (Calculation) or quantitative reasoning problems (Applied Problems). Of the three assessments, Math Fluency is the only test with a time limit. A recent review paper highlights the evidence demonstrating that math assessments with time constraints lead individuals with high math anxiety to perform more poorly than when math assessments do not have time constraints [47]. For example, Faust and colleagues [48] found effects of math anxiety when participants completed mental arithmetic problems under time pressure, but not when they completed the same problems on paper without a time limit. Our Math Fluency assessment required students to mentally solve or recall as many simple arithmetic problems as they could within three minutes, while switching between addition, subtraction, and multiplication. Although this assessment included the least complex problems of the three different tests, the added time pressure likely made this assessment the most anxiety provoking. It is possible that other aspects of math such as complex calculations and word problems would also elicit greater math anxiety if they had to be solved under time pressure.

### ANS acuity and math performance

We also found differential associations between ANS acuity and math ability depending on the specific math skill being measured, reflecting some of the inconsistent findings regarding the association between ANS acuity and math ability reported in the literature (for review, see [49]). Students’ ANS acuity did not correlate with their ability to perform mathematical computations in traditional written format (Calculation) but did correlate with their ability to perform speeded mental arithmetic (Math Fluency) and quantitative reasoning problems (Applied Problems).

Together, the Calculation and Math Fluency findings are in line with a recent meta-analytic review of studies with children and adults: ANS acuity was more strongly correlated with performance on mental arithmetic compared to written arithmetic [50]. Our Calculation assessment required students to perform computations on traditionally written problems (e.g., long division, subtraction with borrowing, multiplying decimals, dividing fractions). In written computations, problems are often solved through a series of learned algorithms in which intermediate operations are computed digit by digit (e.g., addition with regrouping; subtraction with borrowing; long division) without deep processing of the magnitude of the operands or of the solution [51–53]. Thus, these skills should depend more on differences in instruction, recency of instruction, and amount of instruction and practice, rather than differences in numerical magnitude understanding. Indeed, the only significant predictor of students’ calculation scores in our study was the number of college math courses they had taken.

In contrast, the Math Fluency problems assessed the ability to solve speeded mental arithmetic problems. Importantly, ANS acuity remained a unique significant predictor of Math Fluency scores after controlling for the number of college math courses taken, math anxiety, and working memory. In mental computations, students evoke the magnitude of the operands, quickly estimate the proximity between, and use this information to choose the most efficient mental calculation strategy (e.g., starting with the larger addend, solving subtraction with indirect addition) [51–53]. On the Math Fluency assessment, a sharp ANS acuity would likely help students choose efficient mental calculation strategies and flexibly switch between operations, as the assessment requires rapid switching between addition, subtraction, and multiplication [51–53].

In our study, ANS acuity also correlated with Applied Problems, over and above the number of college math courses taken, math anxiety, and working memory. These findings are in line with those of Libertus and colleagues [54] who found adults’ ANS acuity to correlate with their quantitative scores on a college entrance exam, which requires knowledge of arithmetic, geometry, algebra, and data analysis and which frequently presents questions in word problem format or in an applied context. The Applied Problems assessment used in this study required students to listen to orally presented word problems with some visual aids, select only the relevant information, choose the correct operation, transform the information into math equations, and use an efficient strategy to solve the problem. Reasoning through a word problem to determine the correct operation while ignoring the irrelevant information requires a deep understanding of the magnitudes presented in the problem, and even more importantly the relations between the magnitudes. People with more precise ANS representations may be more likely to choose more efficient magnitude-based strategies to solve the problem [55] and to monitor magnitude errors they may have made in the solution [25,54,56]. We note that this potential explanation relies on the assumption that multi-digit numbers are processed holistically, and acknowledge that the exact nature of multi-digit number processing is still debated [57–61].

In addition to helping people make intuitive estimates about the number of items in a collection, the ANS also helps people understand the ordinal relationship between numbers [62] which may translate into an intuitive understanding of addition as ‘getting bigger’ in number and subtraction as ‘getting smaller’ in number [54]. Thus, the ANS likely facilitates online error detection when performing math, such that people with more precise ANS representations can more easily detect erroneous symbolic answers that are beyond the range of likely outcomes (e.g., realizing that the solution did not ‘get bigger’ in number when performing addition) [25,56]. Detecting these types of errors would be most noticeable on the Applied Problems assessment where there is a risk of selecting the incorrect operation (unlike the Calculation or Math Fluency test) and there is sufficient time allotted per problem to check solutions (unlike the Fluency test).

The relation between ANS acuity and Applied Problems was further qualified by the significant interaction with math anxiety. Thus, it appears that the relation between ANS acuity and performance on Applied Problems changes with varying levels of math anxiety: The positive association between ANS acuity and Applied Problems is only present in students with high math anxiety. For these students, ANS acuity may serve as a protective factor that boosts performance to the level of their low math anxious peers. According to one account of the relation between math anxiety and math performance, people with high levels of math anxiety have difficulty focusing on the math task because the worrisome thoughts consume their cognitive resources [12]. If highly math anxious students are more prone to making mistakes because of limited working memory and attentional resources, ANS acuity may play an important role in detecting these mistakes on the Applied Problems test where this type of error detection is possible [25,54,56]. In the case of Applied Problems, our results suggest that differences in math performance relate to the unique combinations of ANS acuity level with math anxiety level, rather than a meditational pathway from ANS acuity to math anxiety to math performance [29].

### Limitations and conclusions

There are some limitations of this study that warrant discussion. First, although we administered the math anxiety questionnaire after the math assessments to minimize the influence of the questionnaire on the participants’ math performance, it is possible that the math assessment influenced participants’ responses on the questionnaire. Follow-up experiments that manipulate task order are needed to determine whether task order has an influence on participants’ scores on these measures and the overall pattern of results. Second, given the correlational nature of our study with adults, we cannot make causal claims about the development of ANS acuity and math anxiety and their influence on math performance. Future longitudinal studies examining the development of ANS acuity and math anxiety will help to unravel their association and joint influences on math performance over time. Studies of this nature are especially needed to examine changes in the relations between math anxiety, ANS acuity, and math performance as children reach higher levels of mathematics, begin to internalize gender and racial stereotypes, and self-select math courses and college majors. Future studies should also examine children’s math performance in stressful, ecologically valid testing situations, such as the classroom.

We conclude that both the unique effects and the interaction of ANS acuity and math anxiety on mathematical performance depend on the type of math skill being measured. The occurrence of an interaction between ANS acuity and math anxiety in the context of one mathematical skill, i.e., the ability to solve applied math problems, promotes the idea that ANS acuity and math anxiety should be considered in tandem when examining their respective influences on math abilities.

## Supporting information

S1 Table Pearson correlations between the average accuracy score for each of the four ANS blocks and all other measured variables. (DOCX)

S1 File Data. (XLSX)
