# Supplementary material for: When approximate number acuity predicts math performance: The moderating role of math anxiety
Source: PLoS One. 2018 May 2;13(5):e0195696. doi: 10.1371/journal.pone.0195696 (PMC5931636; doi:10.1371/journal.pone.0195696)
Supplement: S1 Table — (DOCX) [file pone.0195696.s001.docx]

|  | **1** | **2** | **3** | **4** | **5** | **6** | **7** | **8** | **9** |
| --- | --- | --- | --- | --- | --- | --- | --- | --- | --- |
| **1. ANS Block 1** | ⎯ |  |  |  |  |  |  |  |  |
| **2. ANS Block 2** | .39** | ⎯ |  |  |  |  |  |  |  |
| **3. ANS Block 3** | .42** | .36* | ⎯ |  |  |  |  |  |  |
| **4. ANS Block 4** | .38** | .49** | .55** | ⎯ |  |  |  |  |  |
| **5. Math Courses** | .12 | -.21^†^ | -.13 | -.21^†^ | ⎯ |  |  |  |  |
| **6. WM Span** | .13 | .18 | .12 | .15 | -.27* | ⎯ |  |  |  |
| **7. Math Anxiety** | -.17 | -.08 | .05 | .05 | -.09 | -.13 | ⎯ |  |  |
| **8. Calculation** | .08 | .00 | -.08 | .04 | .26* | .14 | -.19^†^ | ⎯ |  |
| **9. Math Fluency** | .29* | .17 | .16 | .20^†^ | -.06 | .22* | -.27* | .26* | ⎯ |
| **10. Applied Problems** | .21^†^ | .16 | .24* | .18 | .06 | .21^†^ | -.22^†^ | .48** | .40** |

*Note.* ^†^*p*<.10. **p*<.05. ***p*<.001.
